# A serial mediation model reveals the association between parental over-protection and academic entitlement among nursing students

**DOI:** 10.1038/s41598-024-66207-6

**Published:** 2024-07-08

**Authors:** Biao Peng, Ningning Hu, Hong Li, Bing Pang, Mingzhi Lv, Xiuhong Wang, Yufeng Li

**Affiliations:** 1 School of Marxism, Changsha Social Work College, Changsha, China; 2 School of Nursing, Xinjiang Medical University, Urumqi, China; 3 School of Nursing, Guizhou Medical University, Guiyang, China; 4 School of Nursing, YunNan Economics Trade and Foreign Affairs College, Kunming, China; 5 The Second Affiliated Hospital of Xinjiang Medical University, Urumqi, China

**Keywords:** Psychology, Human behaviour

## Abstract

The study aims to explore the influence of parental over-protection on academic entitlement of nursing students, and examine the mediating roles of external locus of control and psychological entitlement. The study sampled two medical universities in Guizhou and Yunnan provinces, China. Participants were nursing students in grades one to four (N = 1003; mean age = 19.51 years; 81.95% female). Using a structural equation model, we examined the mediating effect of external locus of control and psychological entitlement on parental over-protection and academic entitlement. The results show that there was a significant correlation between all variables, and external locus of control and psychological entitlement played a serial mediating role between parental over-protection and academic entitlement. Our findings suggest that academic entitlement of nursing students can be reduced by adjusting parental rearing behaviors, reducing students’ psychological entitlement, and teaching them how to form a healthier attribution style.

## Introduction

Academic entitlement is an unhealthy psychological characteristic first proposed by Dubovsky after observing medical students^1^. It can be generally defined as students’ subjective belief that they are qualified for academic success without contributing individual effort^2^. Researchers regarded academic entitlement as psychological entitlement until Morrow proposed that academic entitlement and psychological entitlement are different psychological structures^3^. Academic entitlement is a privilege consciousness that only exists in the academic environment, while psychological entitlement is a more broad feeling of holistic privilege, in that one feels they have the right to preferential treatment or exemption from subjective beliefs or social responsibility.

Current research on academic entitlement primarily focuses on two aspects: its negative impacts and influencing factors. On the one hand, studies have shown that high levels of academic entitlement lead to a range of adverse learning outcomes, such as low class participation (Knepp^4^), absenteeism, truancy, and academic dishonesty^5,6^, which can ultimately affect students' mental health and the entire educational system^7^. On the other hand, the main factors influencing the development of academic entitlement include demographic variables (e.g., gender^8^, age and work hours^9^), personality traits (e.g., Locus of control^8^, dark triad traits^10^), and parenting styles (e.g., permissive parenting and authoritarian parenting^6,11^). While existing research has primarily focused on the negative impacts of academic entitlement on students and society, few studies have explored the psychological mechanisms underlying the development of academic entitlement. Furthermore, most existing studies have focused on the academic entitlement of non-medical students, with limited attention given to nursing students. However, high levels of academic entitlement among nursing students can have more significant negative impacts on their future nursing skills and overall quality of care. For instance, their shallow learning and knowledge may lead to a lack of in-depth theoretical understanding and weaker practical skills^12^**.** This, in turn, can result in inadequate professional competencies among nursing students. Ultimately, such deficiencies can affect the overall quality of their future nursing skills and compromise patient safety^12^. Therefore, it is of great practical significance to explore the influencing mechanism of academic entitlement of nursing students.

### Parental over-protection and academic entitlement

According to Bronfenbrenner's ecological systems theory, family, as a microscopic system, has the most direct influence on an individual’s psychological development and, in this system, parenting style plays an extremely important role^13^. Parenting style refers to the synthesis of parents’ stable attitudes and beliefs related to parenting in the relatively stable cross-context of parent–child communication^14^. As children typically share the closest relationships with their parents, parenting styles can significantly shape children's behavior, attitudes, cognition, emotion, and overall development^15,16^. Arrindell et al. divided parenting styles into three dimensions: emotional warmth, rejection and over protection^17^. Parental over-protection, also known as excessive control or low autonomy grant, refers to the control imposed by parents to restrict or threaten children's autonomy^18^. Self-determination theory is a theory of human motivation concerning people's self-determined behavior, positing that autonomy is one of the basic psychological needs of human beings^19^. According to self-determination theory, Parental over-protection restricts children's autonomous space, excessively controls and intervenes in children's behaviors, hindering the fulfillment of the need for autonomy, and thereby reducing children's intrinsic learning motivation^20,21^. Simultaneously, over-protective parents tend to impose overly strict demands and monitoring on their children's learning, causing children's learning to be more driven by external expectations rather than intrinsic motivation^22^. Such external pressure and control also inhibit the development of intrinsic learning motivation^23^. Children lacking intrinsic motivation are more likely to view learning as an added obligation, develop excessive expectations for their academic performance, and consequently develop an academic entitlement attitude^24^.

Previous meta-analyses have demonstrated a significant negative correlation between parental over-protection and academic achievement^25^. Fletcher et al. further found that over-protective parenting styles positively influence academic entitlement^26^. Although existing studies have shown empirical support for the influence of parental over-protection on academic entitlement, the internal mechanism of academic entitlement has not been explored deeply. Therefore, this study introduced two variables—external control of the locus of control and psychological entitlement—to investigate the influencing mechanism of parental over-protection on academic entitlement in nursing students.

### Mediating role of external control

To further Heider and Weiner’s attribution theory, Rotter introduced the theory of “source of control”^27^. The locus of control refers one’s attribution of event or behavior results on their surrounding environment, noting two extremes as control sources: internal and external^27,28^. Students with high external control will attribute their academic performance to external factors such as luck, opportunity, and social environment, and believe that their own work hard will not fundamentally change their learning results. Therefore, they place strict demands on teachers and schools as being responsible for them achieving well academically. Previous studies have shown that external control significantly predicts academic entitlement^29–31^. The formation and development of locus of control is not greatly influenced by congenital genetics; rather, the family environment has been shown to be the main influencing factor^32^. In fact, previous studies have found that parental over-protection had a significant effect on college students' external control^33,34^. The overprotective parenting style over-controls the a child’s daily behaviors and activities, restricting the children for long periods of time and conditioning them to listen to others’ opinions rather than forming their own, thus forming a high sense of external control^35^. Previous studies have shown that parental over-protection is positively correlated with a child’s sense of external control^36,37^, external control significantly predicts academic entitlement^29–31^, parental over-protection has a positive impact on academic entitlement^26^. Therefore, external control may play a mediating role in the impact of parental over-protection on academic entitlement.

### Mediating role of psychological entitlement

Psychological entitlement refers to an individual’s sustained, stable, and subjective belief that they have the right to receive preferential treatment^38^. Highly psychological entitlement individuals exhibit heightened sensitivity to fairness concerns and believe they should receive greater "rewards" while exerting minimal effort^39^. In the academic domain, those high in psychological entitlement are more likely to engage in negative behaviors such as academic misconduct^10,40^. College students regard themselves as “consumers” in the university setting due to paying college fees, and many will hold the view that they should be able to complete their studies smoothly, “with less effort and the least [amount of] unpleasant [experiences]”^1^. Students with high level of psychological entitlement, then, are more likely to also exhibit academic entitlement.

However, psychological entitlement is not an innate trait, rather it is influenced by situational and individual factors^41^. Parenting style is one of the important situational factors, which plays an important role in the generation of one’s psychological entitlement. This can be explained through self-determination theory, which posits that humans have three basic psychological needs: autonomy, relatedness, and competence^19^. When these needs are satisfied, individuals experience positive developmental outcomes. Conversely, if these needs remain chronically unmet, individuals may develop compensatory motives to fulfill these needs through alternative means^19,42^. Based on self-determination theory, negative parenting styles (such as excessive control, lack of attention and support) fail to meet children's basic needs for autonomy, relatedness, and competence. To compensate for these deficits, children may develop a sense of psychological entitlement, believing they deserve more privileges and preferential treatment than others as a means to satisfy their deprived basic needs^43,44^.

In addition, parental over-protection leads to the child developing an unrealistic self-concept, drawing excessive attention to themselves, thinking that their own needs are more important than those of others, and taking for granted what they receive as well as what others sacrifice, all of which are key indicators of psychological entitlement^8,43,45^. Previous literature shows that an over-protective parenting style will enhance one’s psychological entitlement^46^ as well as relational entitlement^47^. In sum, parental over-protection has a positive impact on both academic and psychological entitlement, and psychological entitlement itself has a positive impact on academic entitlement. Therefore, psychological entitlement may play a mediating role in the influence of parental over-protection on academic entitlement.

### The relationship between external control and psychological entitlement

According to the above literature review, we understand that external control and psychological entitlement play mediating roles in the relationship between parental over-protection and academic entitlement. However, in the influence of parental over-protection on academic entitlement, the relationship between external control and psychological entitlement merits further investigation. According to attribution theory^48^, the sense of external control of individual locus of control is closely related to psychological entitlement. People with high external control have lower expectations, believing that they are unable to affect any reward. They also do not see the relationship between effort and reward, and instead believe that success comes from what others give. This results in them opting not to proactively strive or fight for achievements or rewards, thereby reinforcing their sense of psychological entitlement. Studies have shown that locus of control is an antecedent variable of psychological entitlement^49,50^. Therefore, external control and psychological entitlement may play a serial mediating role in the influence of parental over-protection on academic entitlement.

### The present study

**Figure 1:**
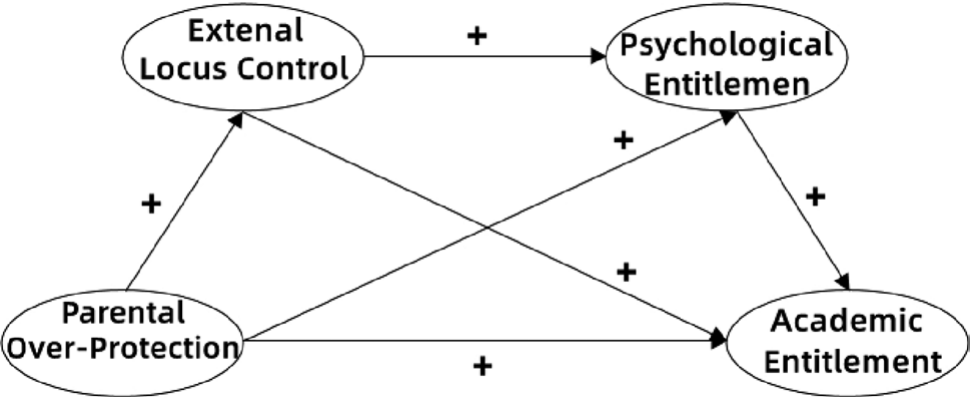
Conceptual model.

In consideration of the existing findings, then, this study took into account the external locus of control and psychological entitlement in considering the model of the influence of an over-protective parenting style on the academic entitlement of college students, and constructed a conceptual model to explore the influence mechanism of parental over-protection on the academic entitlement of nursing students, and proposed the following hypotheses: 1. The external locus of control of nursing students mediates the relationship between parental over-protection and academic entitlement; 2. Psychological entitlement of nursing students mediates the relationship between parental over-protection and academic entitlement; 3.The external locus of control and psychological entitlement of nursing students play a serial mediating role in the influence of parental over-protection on academic entitlement (Fig. 1).

## Methods

### Participants

A cross-sectional descriptive study was conducted between March and June 2021 to clarify the relationships between parental over-protection, external locus of control, psychological entitlement, and academic entitlement among nursing students. A convenience sampling method was used to select participants. The participants used in this study were recruited from two universities in Guizhou and Yunan Provinces, China. A total of 1003 nursing students were recruited to complete the questionnaires. Among them, participants were aged 17 to 25 years old (*M* = 19.51, *SD* = 1.60), 181 (18.05%) were male and 822 (81.95%) were female, 475 (47.36%) were freshman, 245 (24.43%) were sophomore, 217 (21.64%) were junior, 66 (6.58%) were senior.

### Ethical approval

This study was approved by the ethics committee of Guizhou Medical University. Before the investigation, we consulted with the local education department and the investigating school and obtained their approval. After obtaining informed consent from the participants, the questionnaire was completed in a classroom setting. The participants were informed about the purpose of the study and, prior to the start of the study, were assured that their privacy would be protected. All methods in this study were performed in accordance with the ethical standards of the institutional and/or national research committee and the 1964 Helsinki declaration and its later amendments.

### Data collection procedure

Before the investigation, we consulted with the local education department and the investigating school and obtained their approval. After obtaining informed consent from the participants, the questionnaire was completed in a classroom setting. A total of 1058 questionnaires were received. Responses were screened using the following criteria to ensure data quality: (1) Incomplete responses: Questionnaires with more than 3 items missing were excluded. (2) Straight-lining: Questionnaires showing a consistent pattern of the same response option being selected across a full measure were removed. After screening, 1003 valid and attentive responses remained, yielding an effective response rate of 94.80%..

### Instruments

#### The Short Egna Minnen Barndoms Uppfostran (S-EMBU)

The original The Short Egna Minnen Barndoms Uppfostran (S-EMBU) was translated by Jiang et al.^51^ to be used in Chinese college students. The questionnaire is divided into two, one part for the father and one part for the mother. Each of the two questionnaires contains 21 identical items which cover rejection (six items), emotional warmth (seven items), and parental over-protection (eight items). The measure uses a four-point Likert scale, from 1 (No, never) to 4 (Yes, most of the time). Question 17 is a reverse-scored question. A high score in any of these dimensions on either the father or mother questionnaire indicates that individual’s particular style of parenting. The parenting style of each parent can be the same or different. In this study, according to the research hypothesis, we used only the parental over-protection dimension of the questionnaire. In the current study, the Cronbach’s α for the paternal and maternal over-protection questionnaires were 0.71 and 0.72, respectively.

#### The academic entitlement questionnaire

The academic entitlement questionnaire was compiled by Kopp et al.^2^ and translated by Hu et al.^52^ for use among Chinese college students. The questionnaire consists of eight items with a single dimensional structure. The measure uses a seven-point Likert scale, from 1 (strong disagreement) to 7 (strong agreement). The higher the total score, the stronger one’s level of academic entitlement. In the current study, the Cronbach’s α of the Chinese version of the AEQ was 0.73.

#### The Psychological Entitlement Scale (PES)

The Psychological Entitlement Scale (PES) was developed by Campbell et al.^38^ and translated by Bai and Wang^53^ for use among Chinese college students. The scale consists of nine items with a single dimensional structure, and uses a seven-point Likert scale, from 1 (strongly disagree) to 7 (strongly agree). The higher the total score, the stronger one’s level of psychological entitlement. In the current study, Cronbach’s α = 0.89.

#### The Locus of Control Scale (LCS)

The Locus of Control Scale (LCS) was compiled by Levenson et al.^54^ and translated by Xiao and Chen^55^ for use among Chinese college students. The scale is composed of three parts: internal control, authority, and opportunity. The two dimensions of authority and opportunity reflect external locus of control. Each dimension has eight items, for a measure total of 24 items. Each item is rated on a six-point Likert scale from 0 (strongly disagree) to 5 (strongly agree). The higher the total score, the higher one’s tendency towards that particular dimension. Only the authority and opportunity dimensions were used in the current study, and the Cronbach's α coefficients of authority and opportunity were 0.83 and 0.76, respectively.

### Statistical analysis

First, Harman single factor test was used to test common method bias (CMB)^56^. An exploratory factor analysis with all items and no rotation was conducted using SPSS 25.0. Common method bias was not apparent if the first factor accounted for less than the critical value of 40% of the total variance^56^.

Second, descriptive statistics and correlation analyses of parental over-protection, academic entitlement, psychological entitlement, and external locus of control were performed using SPSS 25.0. *p* < 0.05 (two-tailed test) was considered to be statistically significant.

Third, to verify the mediating effects of psychological entitlement and external locus of control on the relationship between parental over-protection and nursing academic entitlement, structural equation modeling using Amos 23.0 software was used with maximum likelihood estimations. Multiple fit indices were used to evaluate the model, including the chi-square/degrees of freedom ratio (χ^2^/*df*), the standardized root mean square residual (SRMR), the root mean square error of approximation index (RMSEA), the goodness-of-fit index (GFI), the adjusted goodness- of-fit index (AGFI), the normed fit index (NFI), the comparative fit index (CFI), and the Tucker-Lewis Index (TLI). In the present study, the following criteria were used to testify whether the model was fit: χ^2^/*df* < 5.00, SRMR < 0.05, RMSEA < 0.08, GFI > 0.90, AGFI > 0.90, NFI > 0.90, CFI > 0.90, and TLI > 0.90^57^.

## Results

### Common method bias test (CMB)

Exploratory factor analysis resulted in 10 factors with eigenvalues greater than 1. The first factor accounted for 18.46% of total variance, which is less than 40% of the critical standard, which indicated that common method bias was not apparent.

### Descriptive statistics

Table 1 presents participants’ descriptive statistics regarding parental over-protection, academic entitlement, psychological entitlement, authority, and opportunity. The mean score of academic entitlement was 25.68 (*SD* = 6.87). The mean scores of paternal and maternal over-protection were 16.76 (*SD* = 3.50) and 17.55 (*SD* = 3.75), respectively. The correlations among paternal over-protection, maternal over-protection, academic entitlement, psychological entitlement, authority, and opportunity are shown in Table 1. All six variables were significantly correlated.

**Table 1:** Correlations of parental over-protection, academic entitlement, psychological entitlement, authority, and opportunity (n = 1003).

Variable	M	SD	1	2	3	4	5	6
1. Paternal over-protection	16.76	3.50	1					
2. Maternal over-protection	17.55	3.75	0.76**	1				
3. Psychological entitlement	32.26	8.81	0.19**	0.15**	1			
4. Authority	14.85	5.91	0.22**	0.17**	0.35**	1		
5. Opportunity	15.82	5.26	0.20**	0.18**	0.36**	0.76**	1	
6. Academic entitlement	25.68	6.87	0.24**	0.20**	0.45**	0.29**	0.29**	1

**p < 0.01 (two-tails).

### Test of the hypothesized model

**Figure 2:**
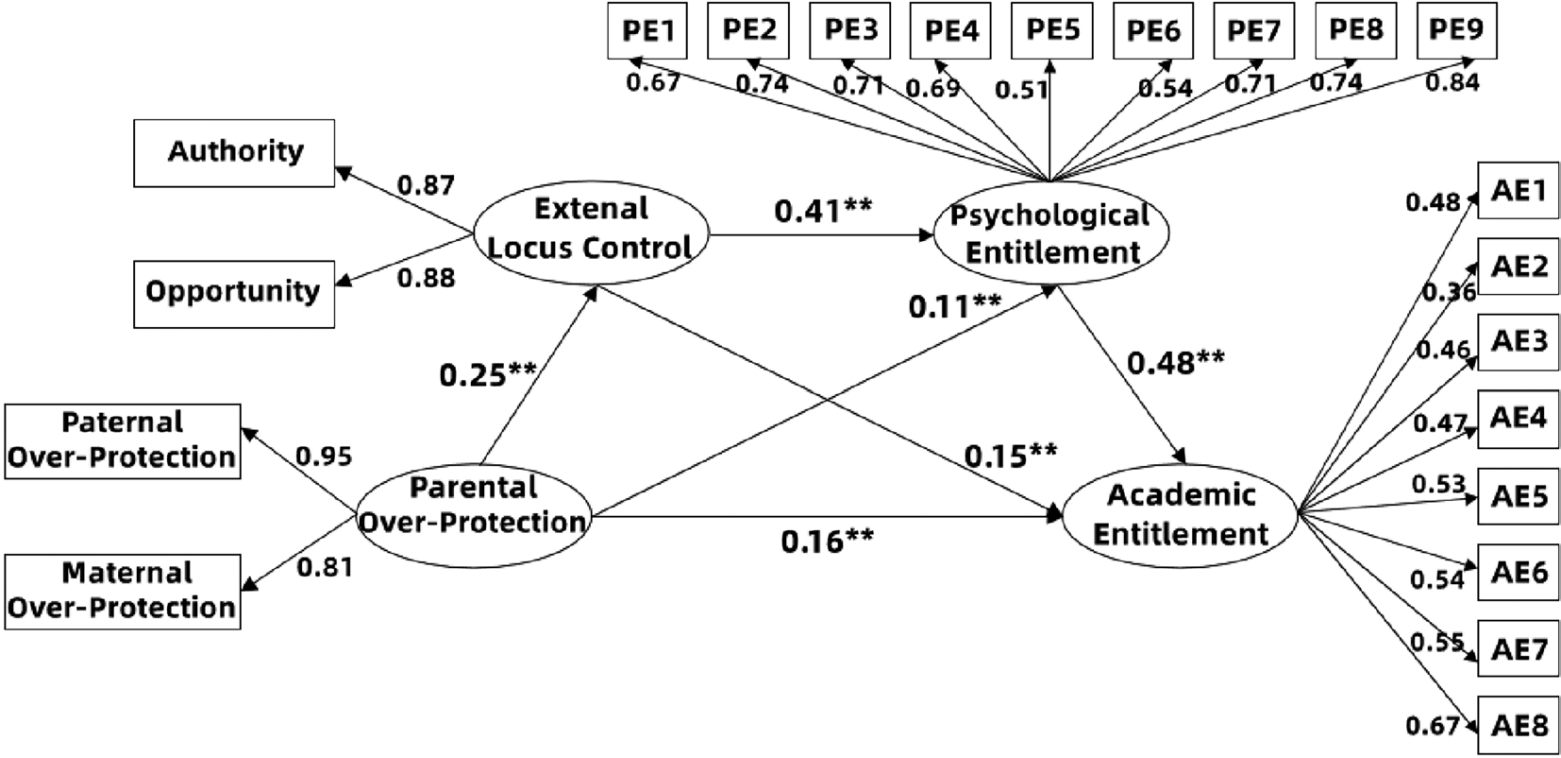
Final model and standardized model paths. **p < 0.01.

The bias-corrected nonparametric percentile Bootstrap method was used to examine the hypothesized model. The fit level of the modeling was satisfactory with CMIN = 864.645, *df* = 183, SRMR = 0.043, RMSEA = 0.06 [90% CI 0.057, 0.065], GFI = 0.91, AGFI = 0.90, NFI = 0.89, CFI = 0.91, and TLI = 0.90. Based on the overall mediating model (Fig. 2 and Table 2), the total effect of parental over-protection on academic entitlement was significant (*β* = 0.29, *p* < 0.01). Parental over-protection positively affected the external locus of control (*β* = 0.25, *p* < 0.01). External control partly mediated the effects of parental over-protection on academic entitlement (*β* = 0.04, *p* < 0.001), which supported Hypothesis 1. Psychological entitlement partly mediated the effects of parental over-protection on academic entitlement (*β* = 0.05, *p* < 0.01), which supported Hypothesis 2. Psychological entitlement and external control play a serial mediating role in the influence of parental over-protection on academic entitlement (*β* = 0.05, *p* < 0.01), supporting Hypothesis 3. The values of total, direct, and indirect effects of the model are shown in Table 2.

**Table 2:** Total, direct, and indirect effects of each model path.

Estimate		B	SE	β	The size of effects	95%CI	
						LLCI	ULCI
Total effect	PO → AE	0.07	0.012	0.29		0.046	0.093
Direct effect	PO → PE	0.03	0.009	0.11		0.011	0.046
	PO → AE	0.04	0.009	0.16		0.019	0.055
	ELC → AE	0.02	0.006	0.15		0.011	0.036
Indirect effect	PO → ELC → AE	0.01	0.003	0.04	12.33%	0.004	0.015
	PO → PE → AE	0.01	0.004	0.05	17.47%	0.005	0.021
	PO → ELC → PE → AE	0.01	0.003	0.05	16.78%	0.007	0.018

*PO* Parental over-protection, *AE* academic entitlement, *ELC* external locus of control, *PE* psychological entitlement, *B* unstandardized coefficients; *β* standardized coefficients.

## Discussion

### Parental over-protection and academic entitlement

The present study found that parental over-protection positively influenced nursing students' academic entitlement, consistent with previous research findings in university student populations^24,26^. This phenomenon can be explained by self-determination theory, which posits that the environment (including the family environment) can enhance people's intrinsic motivation and ensure healthy human development by satisfying the basic psychological need of autonomy, among others^19^. However, an over-protective parenting style has an adverse impact on students' basic psychological needs, thereby affecting their learning motivation and ultimately leading to the formation of academic entitlement^21,23^. Specifically, this may be because over-protection restricts nursing students' autonomy by exerting excessive control and intervention, hindering the fulfillment of their need for autonomy. Simultaneously, this parenting style often imposes excessive external expectations rather than cultivating intrinsic motivation, causing nursing students' learning to be driven more by external pressures. The lack of autonomy and intrinsic motivation diminishes nursing students' proactivity and internal drive for learning. When intrinsic learning motivation is absent, nursing students tend to view academics as an added obligation and develop unrealistically high expectations for their performance, thereby forming a psychological tendency to seek academic entitlement. Therefore, based on self-determination theory, the over-protective parenting style ultimately promotes the formation of academic entitlement by influencing the satisfaction of nursing students' basic psychological needs and the development of their intrinsic motivation. This finding contributes to our understanding of the roots of academic entitlement and provides a theoretical basis for relevant educational interventions.

### Mediating role of external control

The present study also found that external locus of control partially mediated the relationship between parental over-protection and nursing students' academic entitlement. This supported Hypothesis 1. Previous research indicated that parental over-protection undermines children's development of independent consciousness^35,58^. It leads them to believe they cannot determine their own affairs. Instead, they believe they are controlled by mysterious external forces or powerful others. This fosters a high degree of external locus of control. According to attribution theory^48^ and Greenberger et al.^40^, when these children grow into adulthood and enter university, they tend to attribute academic achievements to external sources rather than personal efforts. This occurs if they believe success and failure are driven by factors like luck and teachers, outside their locus of control. Consequently, they are inclined to criticize teachers to obtain better grades and accomplishments. This results in higher academic entitlement. In the current study, this mechanism also applied to nursing students. An over-protective parenting style can instill a sense of external locus of control in them. It leads them to believe academic success is due to luck, opportunity, and power rather than personal effort strongly influencing performance. This further reinforces their psychological academic entitlement tendency. Therefore, it is necessary to pay attention to the nursing students with a tendency towards externalizing their locus of control.

### Mediating role of psychological entitlement

The current study found that psychological entitlement has a partially mediating effect on the relationship between parental over-protection and nursing students’ academic entitlement, confirming Hypothesis 2. Previous studies have in fact shown that an over-protective parenting style causes individuals to pay too much attention to themselves, resulting in an unrealistic self-concept and the belief that they are special and deserve more than others, thus promoting their sense of psychological entitlement^59,60^. This can be explained by self-determination theory: when parents exert excessive control for a prolonged period while lacking attentiveness and support, they fail to meet children's basic needs for autonomy, relatedness, and competence^19^. To compensate for these unmet needs, children may develop a compensatory sense of psychological entitlement, believing they should receive more privileges and preferential treatment than others, thus attempting to satisfy their deprived basic needs through this mindset^19,42–44^. In other words, psychological entitlement is a compensatory motive arising from chronic deprivation of basic psychological needs. This phenomenon also applies to nursing students: when deprived of autonomy, intimate relationships, and competence during their upbringing, they may develop a distorted belief that they deserve more privileges (psychological entitlement) to compensate for their unmet basic needs, thereby maintaining self-esteem and achieving a subjective sense of fulfillment. This irrational belief of psychological entitlement can render students more narcissistic, leading to lower social responsibility and increased propensity for negative behaviors such as academic dishonesty^61^. Academic dishonesty is indeed a significant manifestation of academic entitlement. Therefore, psychological entitlement can positively influences academic entitlement. Additionally, nursing students with high psychological entitlement think that being a nurse means that they are only responsible for injections, infusions, and dispensing of medicine, and that deeper learning of theoretical knowledge and operational skills are therefore useless, creating a fertile environment for academic entitlement and dishonesty^12^.

### The relationship between external control and psychological entitlement

The current study also confirmed that a sense of external locus of control and psychological entitlement have a serial mediating effect in the relationship between parental over-protection and academic entitlement (i.e., parental over-protection—external control—psychological entitlement—academic entitlement), meaning that Hypothesis 3 was also valid. The above studies have shown that the external locus of control and psychological entitlement play mediating roles between parental over-protection and nursing students' academic entitlement respectively. The confirmation of hypothesis 3 shows that the external locus of control affected psychological entitlement, and parental over-protection affected the academic entitlement of nursing students through the external locus of control and psychological entitlement. This is in line with the findings of previous studies, such as Carnes and Knotts^49^ who found that locus of control significantly predicted psychological entitlement. Nursing students with a high external locus of control are unable to see the relationship between effort and reward, and instead only believe that they should be rewarded without considering the impact of their own behavior on outcomes, believing that good outcomes are for others to provide, rather than seeing they should act to persue these good outcomes. Nursing students with a high sense of external locus of control thereby nurture the formation of psychological entitlement. This shows that nursing students’ sense of external control and psychological entitlement further impact the way that parental over-protection impacts nursing students’ academic entitlement. An over-protective parenting style can also indirectly affect nursing students’ academic entitlement by affecting the individual’s sense of external control and psychological entitlement. Therefore, the mediating effect of external control and psychological entitlement provides a new perspective for reducing the academic entitlement of nursing students.

To summarize, the current study supports the theoretical model that an over-protective parenting style influences nursing students’ academic entitlement through individual factors (i.e., sense of external locus of control and psychological entitlement). The results of this study therefore lead to the following suggestions: 1. Establish a collaborative parenting mechanism between families and schools to guide parents in reducing over-protective parenting practices. Parents should loosen their control appropriately, grant nursing students more autonomy, while providing understanding, care, and support, which is conducive to the healthy physical and psychological development of nursing students. 2. Reduce the psychological entitlement of nursing students and guide them in learning to view their own efforts and gains rationally. For example, nursing students’ psychological entitlement can be reduced by enhancing egalitarian concepts, reducing self-compassion, and changing subjective cognition^41^. 3. Nursing students should be taught how to have an objective understanding of themselves, enabling them to form a healthier sense of internal control attribution, and teaching them to believe that academic success or failure depends primarily on their own ability and effort, allowing them to be better able to face learning difficulties positively and optimistically. For example, attribution training could be carried out as group development, intensive corrections, and observational learning^62^. 4. Universities should also prioritize psychological health education for nursing students, assisting them in establishing a proper understanding of academic entitlement, cultivating a sense of social responsibility, and preventing and reducing academic misconduct.

Theoretically, this is the first study to associate over-protective parenting, individual factors (i.e., external locus of control and psychological entitlement), and academic entitlement among nursing students, providing a new perspective for understanding the mechanism underlying nursing students' academic entitlement. Practically, the findings of this study provide targeted intervention suggestions for universities and families, which can help promote the physical and mental health development of nursing students. However, this survey had several limitations. First, the study used the method of convenient sampling investigation into two schools, which may have caused selection bias and may have also limited the generalizability of our research findings. The sample size of this study is somewhat small, which cannot completely represent all nursing students in China. In the future, a large sample survey can be used to test the results of this study. Furthermore, data were collected via self-report questionnaires, which could have led to bias in overestimated responses of the participants. Finally, this study was a cross-sectional study, not cause-and-effect. The causal relationships between the variables require further investigation and verification by combining experiments and performing follow-up studies, so as to further clarify the mechanisms between variables in action.

## Conclusion

The results show that there is a certain degree of academic entitlement in nursing students in China. Furthermore, they verify that perceived external control and psychological entitlement mediate the relationship between parental over-protection and academic entitlement in nursing students. Parental over-protection can not only lead directly to the academic entitlement of nursing students, but it can also indirectly affect the academic entitlement of nursing students through individual factors (e.g., sense of external control and psychological entitlement). Based on these results, we suggest that educators pay attention to the academic entitlement of nursing students. An over-protective parenting style, an externally controlled attribution style, and psychological entitlement are the key factors which can lead to academic entitlement. This study provides a theoretical basis for the intervention of academic entitlement in nursing students in particular.

## Data Availability

The data result are available from the corresponding author on reasonable request.
